# Different strategies in de-escalation of axillary surgery in node-positive breast cancer following neoadjuvant treatment: a systematic review and meta-analysis of long-term outcomes

**DOI:** 10.1007/s12282-025-01692-9

**Published:** 2025-04-05

**Authors:** Vivian Man, Jiaxu Duan, Wing-Pan Luk, Ling-Hiu Fung, Ava Kwong

**Affiliations:** 1 Division of Breast Surgery, Department of Surgery, The University of Hong Kong Li Ka Shing Faculty of Medicine, Queen Mary Hospital, Hong Kong, Hong Kong SAR; 2 The University of Hong Kong Li Ka Shing Faculty of Medicine, The University of Hong Kong Li Ka Shing, Hong Kong, Hong Kong SAR; 3 Medical Physics and Research Department, Hong Kong Sanatorium and Hospital, Hong Kong, Hong Kong SAR; 4 Daniel CK Yu Professor in Breast Cancer Research, Chief of Breast Surgery Division, Department of Surgery, The University of Hong Kong Li Ka Shing Faculty of Medicine, Queen Mary Hospital, Hong Kong, Hong Kong SAR

**Keywords:** Breast cancer, Neoadjuvant chemotherapy, Sentinel lymph node biopsy, Targeted axillary dissection

## Abstract

**Purpose:**

Different surgical options existed in the management of axilla among breast cancer patients who were initially node-positive and were converted node-negative after neoadjuvant systemic treatment (NST). De-escalation of axillary surgery was feasible, but previous studies focused on the false-negative rate (FNR) of respective procedures. The aim of this study is to evaluate the oncological outcomes of sentinel lymph-node biopsy (SLNB), MARI procedure, and targeted axillary dissection (TAD).

**Patients and methods:**

PubMed, Embase, and the Cochrane library literature databases were searched systematically. Studies were eligible if they addressed the axillary recurrence rate of patients with nodal pathological complete response (pCR) and omission of axillary lymph-node dissection (ALND) after NST. Pooled analysis was performed using inverse variance methods for logit transformed proportions.

**Results:**

Eleven retrospective studies and three prospective studies involving 4268 patients with node-positive breast cancers were included. A total of 1650 patients achieved nodal pCR and avoided ALND, 1382 patients with SLNB only and 268 patients with MARI/TAD. The pooled estimate of axillary recurrence was 2.1% (95%CI 1.4–3.2%) for patients with negative SLNB and 1.5% (95% CI 0.5–4.1%) for patients with negative MARI/TAD. There was no significant benefit of ALND over SLNB in patients with nodal pCR after NST. Pooled estimates of 5-year DFS, DDFS, and OS of SLNB alone were 0.87 (95% CI 0.83–0.90], 0.90 (95% CI 0.88–0.92), and 0.92 (95% CI 0.88–0.94), respectively.

**Conclusion:**

Breast cancer patients who are converted node-negative after NST have extremely low nodal recurrence rate, irrespective of the choice of axillary surgery. Omission of ALND is oncologically safe in patients who have nodal pCR after NST.

**Supplementary Information:**

The online version contains supplementary material available at 10.1007/s12282-025-01692-9.

## Introduction

Over the past decades, the use of NST has expanded to become an option for patients with early operable breast cancer [1]. It allows down-sizing of primary breast tumor and eradication of disease in regional lymph nodes before definitive surgery. Pathological complete response (pCR), which is considered the surrogate marker for improved oncological outcome [2, 3], is in particular more frequent in human epidermal growth factor receptor 2 (HER2) positive and triple negative subtypes [4]. Therefore, there is a growing interest in the omission of ALND in excellent responders, who were converted node-negative after NST.

Different strategies have been suggested in the de-escalation of axillary surgery after NST. Sentinel lymph-node biopsy (SLNB) has been evaluated in several prospective multi-institutional clinical trials, including the SENTINA [5], SN FNAC [6], and ACOSOG Z1071 trial [7]. The comparatively lower nodal detection rate and higher FNR of SLNB after NST have been a practical concern for many clinicians [5–7]. Better patient selection and optimal surgical techniques, including the use of dual tracers [5, 6], the retrieval of ≥ 3 sentinel lymph nodes (SLNs) [5, 6], the use of immunohistochemistry [6], and the removal of clipped abnormal node [7], have been advocated to lower the FNR of SLNB. The MARI procedure, on the other hand, was proposed as an alternative to SLNB by the Netherlands Cancer Institute [8]. An I-125 seed was implanted into the cytology-proven axillary lymph node before the start of NST. These nodes, when excised during definitive axillary surgery, were found to be predictive of axillary status post-neoadjuvant treatment with an FNR of 7%. The added value of selective extirpation of these marked axillary lymph nodes was further demonstrated by Caudle et al. [9], in which the combination of SLNB and clipped node excision (TAD) reduced the FNR to 1.4%.

Despite extensive efforts to find the optimal procedure for axillary de-escalation, there is wide heterogeneity in surgical approaches among international breast surgeons and radiation oncologists [10]. Galimberti et al. [11] found an extremely low axillary failure rate with SLNB alone and suggested minimal clinical prognostic significance with procedural FNR. Their favorable oncological outcome was also supported by recent meta-analysis, which demonstrated high rates of DFS and OS at 5 years among those with negative SLNB alone [12]. Therefore, this systematic review and meta-analysis aim to evaluate the pooled axillary recurrence rate, DFS, DDFS, and OS of axillary de-escalation surgeries among patients who achieved nodal pCR after NST.

## Patients and methods

### Literature search

This systematic review was conducted in accordance with the PRISMA statement. The literature search was conducted using PubMed, Embase, and Cochrane Library up to May 2023. The following free text terms were used to search for relevant literature: (node-positive breast cancer) AND (neoadjuvant chemotherapy) AND (sentinel lymph node biopsy OR targeted axillary dissection). The bibliographies of relevant articles were studied to identify further relevant literature. The resulting titles and abstracts were screened by two independent authors (VM and JD) and relevant articles were retrieved to review the full manuscript for relevance and level of evidence. The last search was conducted on 26th May 2023. Selected studies were appraised and analyzed for relevant data. Corresponding authors of the included studies were contacted by email for relevant unpublished data.

### Inclusion and exclusion criteria

Studies were included if they satisfied the following eligibility criteria: (1) they included patients with node-positive breast cancer who underwent NST, subsequently achieved nodal pCR and avoided ALND; (2) they described the cumulative incidence of axillary recurrence with or without other oncological outcomes; (3) they were clinical cohort studies consisting of a minimum of 20 patients and attaining a satisfactory quality assessment score. Studies that failed to fulfill the inclusion criteria, or did not report on the outcomes of interest, were excluded from the analysis. Reviews, commentaries, conference abstracts, case reports, and non-English articles were also excluded. Duplicated articles and references were identified and removed. If data from the same study were published on different occasions, only the latest version of the data was retrieved for analysis. The primary outcome was the cumulative incidence of axillary recurrence.

### Data extraction

The quality of the included articles was assessed by the Newcastle–Ottawa Scale [13]. Each article was rated on three dimensions, which were patient selection, comparability between cohorts, and outcome analysis. A maximum score of 9 was given to the highest quality studies. Data were then extracted from the selected articles, including study design, method of axillary de-escalation under evaluation and its intraoperative details, number of patients with node-positive breast cancers, number of patients with conversion to clinical and pathological node-negative status after NST, adjuvant radiotherapy in particular the application of nodal field irradiation, duration of follow-up, and survival outcomes. Extracted data were tabulated, and disagreements were resolved by consensus.

### Statistical analysis

Clinicopathological and intraoperative details were presented in tables as descriptive statistics. Axillary recurrence was defined as a recurrence in the ipsilateral level I–III axillary lymph nodes. DFS, DDFS, and OS were calculated from the day of surgery to the date of respective event, death, or last follow-up. Statistical analysis was performed with the R software. Meta-analysis for axillary recurrence, DFS, DDFS, and OS was done with R package {meta}, using inverse variance methods for logit transformed proportions. Individual study results and the pooled estimates were displayed as forest plots with a 95% confidence interval. Statistical heterogeneity among studies was assessed by the Chi-square test for heterogeneity and by calculating the I^2^ statistic. A fixed-effect model was used to calculate the pooled outcome. In case of significant heterogeneity (I^2^ > 50%), a random-effect model was used. P value less than 0.05 was considered statistically significant. Additionally, a Leave-One-Out Sensitivity Analysis has been conducted to examine the sensitivity of the meta-estimations in the analysis of axillary recurrence and other secondary oncological outcomes.

## Results

### Selection of studies

**Fig. 1:**
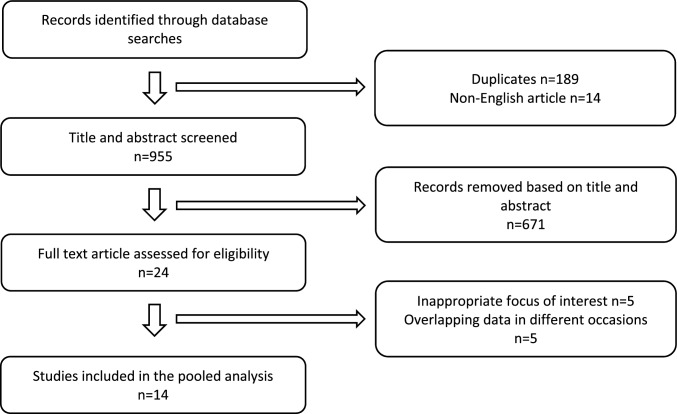
PRISMA flowchart for the study

A total of 1158 articles were identified from PubMed, Embase, and Cochrane Library for title screening. There were 14 non-English and 189 duplicated articles, which were excluded. After screening the titles and abstracts, 260 were reviews, commentaries, or conference abstracts, and 671 were found irrelevant. A total of 24 articles were eligible for full-text review. Among these, the oncological outcome of patients with successful nodal conversion and axillary surgery de-escalation was not clearly described in five studies. Another five articles were excluded for overlapping data published on different occasions [14–18]. As a result, 11 retrospective studies [20–30] and 3 prospective studies [19, 31, 32] matched the inclusion criteria and were included in this meta-analysis (Fig. 1). Detailed information on the reviewed articles is displayed in Table 1.

**Table 1:** Characteristics of included studies

References	Study type	Quality score	Axillary de-escalation evaluated	No of patients	Clinical T staging before NST	Clinical N staging before NST	No of patients with cN + pre-NST	No of patients converted to ycN0
Martelli et al. [19]	Prospective	9	SLNB	353	T2	N0-1	216	216
Barrio et al. [20]	Retrospective	7	SLNB	769	T1-3	N1	610 (cN1)	555
Cabioglu et al. [21]	Retrospective	8	SLNB	303	T1-4	N1-3	303 (256 cN1)	Not specified
Sanchez et al. [22]	Retrospective	7	SLNB	399	T1-4	N0-2	180	180
Kahler-Ribeiro-Fontana et al. [23]	Retrospective	9	SLNB	688	T1-3	N0-2	222	222
Lee et al. [24]	Retrospective	7	SLNB	760	T1-4	N0-3	350	Not specified
Kim et al. [25]	Retrospective	8	SLNB	223	T1-4	N1-3	223	223
Damin et al. [26]	Retrospective	7	SLNB	131	T1-4	N1-3	131	59
Wong et al. [27]	Retrospective	9	SLNB	243	T1-3	N0-2	132	132
Piltin et al. [28]	Retrospective	7	SLNB	602	T1-4	N1-3	602	Not specified
Choi et al. [29]	Retrospective	8	SLNB	506	T1-4	N1-3	506	Not specified
Van Loevezijn et al. [30]	Retrospective	9	MARI	272	Not specified	Not specified	272	Not specified
Kuemmel et al. [31]	Prospective	9	TAD	199	T1-4	Not specified	199	152
Wu et al. [32]	Prospective	8	TAD	322	T1-4	N1-3	322	242

### Study characteristics and quality assessment

The included cohorts consisted of 14 studies published between 2018 and 2023. All studies achieved an overall score of 7 or above on the Newcastle–Ottawa Scale [13] (Table 1). Eleven studies focused on the oncological outcomes of SLNB alone [19–29], while the remaining three articles assessed newer surgical modalities of MARI [30] and TAD [31, 32]. They comprised a total of 4268 patients with node-positive breast cancers before NST.

### Clinical and radiological assessment

Majority of the recruited patients had clinical T1-3 (95.0%) and N1 (78.9%) breast cancers (Table 1). Eleven studies reported on the biological subtypes of primary breast tumors [20, 21, 23, 25–32]. Among these, 39.6% were hormone receptor (HR)-positive and human epidermal growth factor receptor 2 (HER2)-negative tumors; 20.9% were HR-positive and HER2-positive; 16.2% were HER2-positive only and 23.0% were triple negative. Five studies only recruited patients who were converted to clinical or pathological node-negative breast cancers after NST [19, 22, 23, 25, 27]. Patients with inflammatory breast cancers [23, 25, 27–29, 32], other prior malignancies [19, 23, 25, 28, 30], prior ipsilateral axillary surgery [26, 27, 29], or distant metastases at diagnosis [19, 21, 23–29, 31, 32] were largely excluded.

Axillary ultrasonography (AUS) was the most commonly used imaging modality for nodal assessment [19, 21–23, 25–32] (Table 2). In most studies, positive needle biopsy of suspicious axillary lymph nodes was mandatory to confirm nodal metastases [20, 21, 24, 27–32]. Abnormal AUS [19, 22, 25, 26], magnetic resonance imaging (MRI) [22, 25], or positron emission tomography (PET) scan [23] were used otherwise to define clinical node-positive status. After completion of NST, nodal response was reassessed with physical examination only [20, 27] or with the addition of AUS [19, 22, 23, 26, 29–31], MRI [22, 29] or PET scan [23]. Five studies did not specify the mode of clinical assessment before surgery [21, 24, 25, 28, 32]. Therefore, the definition of clinical nodal conversion varied among these studies. A total of 1981 patients (46.4%) achieved clinical nodal complete response after neoadjuvant treatment.

**Table 2:** Preoperative nodal assessment and sentinel lymph-node (SLN) identification

Reference	Pre-NST node assessment	Post-NST node assessment	SLN mapping	SLN identification rate	Median number of nodes excised (range)	No of patients with SLNB only
Martelli et al. [19]	Physical examination and AUS	AUS	Single tracer; 99Technetium labeled radiocolloid	Not specified	2 (1–8)	91
Barrio et al. [20]	Radiological assessment not specified; biopsy-proven	Physical examination	Dual tracer; technetium-99 m sulfur colloid and isosulfan blue dye	(513 with 3 or more SLNs)	(513 with 3 or more SLNs)	234
Cabioglu et al. [21]	AUS, PET and MRI; FNA or biopsy proven	Not specified	Single tracer with blue dye (66%) or combined blue dye and 99Technetium labeled radiocolloid	100	3 (1–6)	303
Sanchez et al. [22]	AUS, MRI or core biopsy CT or PET;	AUS; MRI	Single tracer; Patent blue V or methylene blue	95.2	3 (1–7)	94
Kahler-Ribeiro-Fontana et al. [23]	AUS; FNA or PET	AUS; PET	Single tracer; 99mTechnetium labeled radiocolloid	100	2 (1–6)	132
Lee et al. [24]	FNA or core biopsy; Radiological assessment not specified	Not specified	Single tracer; colloid radioisotope	Not specified	Mean 4.9 SLN	350
Kim et al. [25]	AUS or MRI	Imaging not specified	Mostly single tracer; radioisotope	61.8	Mean 2.2 SLN	94
Damin et al. [26]	AUS (not biopsied)	AUS	Dual tracer; 99mTechnetium labeled radiocolloid and patent blue dye	93.2	Mean 2 SLN	38
Wong et al. [27]	AUS; core biopsy	Physical examination	Dual tracer; 99Technetium labeled radiocolloid and patent blue dye	96.9	4 (3–6)	102
Piltin et al. [28]	AUS; FNA	Not specified	Not specified	96.2	3 (1–12)	159
Choi et al. [29]	AUS; FNA	AUS and MRI	Technetium-99 m sulfur colloid and/or vital blue dye	98.3	5 (2–9)	85
Van Loevezijn et al. [30]	AUS, MRI; PET; FNA	AUS	–	–	–	–
Kuemmel et al. [31]	AUS; Core biopsy	AUS	Technetium-99 m ± blue dye	Not specified	*TAD 3 (1–10)	–
Wu et al. [32]	AUS; Core biopsy or FNA	Not specified	Dual or single tracer; radiolabeled colloid or blue dye	95.8	Not specified	–

*FNA*  fine needle aspiration

### Neoadjuvant systemic treatment

The neoadjuvant chemotherapy regimens were clearly described in seven studies [19–23, 29, 32]. Sixty-four percent of patients received a combination of anthracyclines and taxanes [19–23, 25, 26, 29, 32], in sequential [20–22, 25] or concurrent [19, 22] manner. Anthracycline-alone or taxane-alone treatment was less common [23, 24, 29]. Carboplatin was used in 10.5% of patients, mainly in triple negative diseases in several studies [20, 21, 32]. Anti-HER2 treatment was added in patients with HER2-positive breast cancers, which constituted around 35.3% of patient population, in the form of trastuzumab alone [19, 21–23, 26, 29, 32] or trastuzumab/pertuzumab combination [20, 21, 32].

### Intraoperative axillary nodal surgery

Among the 11 studies that evaluated SLNB, five studies used a single localizing tracer with radiocolloid [19, 23–25] or blue dye [22] (Table 2). Three studies adopted the dual tracer localization technique [20, 26, 27]. A high sentinel lymph-node localization rate of > 90% was reported in seven studies [21–23, 26–29] regardless of the localization method. On the contrary, Kim et al. [25] found an extremely low success rate of 61.8% with single tracer radioisotope localization, in which 58 patients received ALND due to failed mapping. Six studies reported a median or mean of 3 or more SLNs harvested during operation [21, 22, 24, 27–29]. In the series published by Barrio et al., 3 or more SLNs were retrieved with dual tracer localization in 92% of patients undergoing SLNB [20]. Those with inadequate sentinel lymph-node sampling were managed with ALND.

Radioactive iodine seed was used to mark the largest pathology-proven tumor-positive axillary lymph node prior to the start of NST in the MARI procedure [30]. Selective removal of the MARI node was performed in definitive surgery with the use of gamma probe. The median number of axillary lymph nodes excised was one (range 1–6). The MARI node was not identified in 2% of patients. Similarly, Kuemmel et al. [31] and Wu et al. [32] clipped the biopsied-proven axillary lymph node prior to NST. Wire localization of the clipped lymph node under ultrasound guidance was performed together with single or dual tracer localization of SLNs during definitive surgery. Mammographic localization of the clipped axillary lymph node could be an alternative. The median number of TAD lymph nodes was 3 (range 1–11) [31]. TAD was attempted but unsuccessful in around 10% of both cohorts [31, 32], due to failed sentinel lymph-node mapping or failed localization of the clipped node. Among these 14 studies, a total of 1650 patients achieved nodal pCR and avoided ALND (Table 3).

**Table 3:** Pathological nodal results and oncological outcomes in SLNB-alone group

Reference	No of patients with ypN0 and avoid ALND	No of patients with ypNi + /mi and avoid ALND	No of patients with ypN0 and ALND	Adjuvant nodal field radiotherapy to ypN0	Median follow-up period, months (range)	Axillary recurrence in ypN0 and no ALND (%)	Axillary recurrence in ypN0 and ALND
Martelli et al. [19]	81	Not specified	40	No	87 (48–112)	0/81 (0)	0/40 (0)
Barrio et al. [20]	234	Not specified	–	Yes (70%); level I-III, SCF, IMC#	40 (2.3–76)	1/234 (0.4) (refused radiotherapy)	–
Cabioglu et al. [21]	211	52	–	Yes (100%); level I-III, SCF ± IMC	36 (24–172)	0/211 (0)	–
Sanchez et al. [22]	86	8	–	No	35.6 (2–55)	4/86 (4.7)	–
Kahler-Ribeiro-Fontana et al. [23]	123	8	–	Only 23.4% of the whole cN1/2 cohort; part of level II, III, SCF	9.2 year (IQR 5.3–12.3)	ypN0 2/123 (1.6) ypNi + /mi 2/8 (25)	–
Lee et al. [24]	242	Not specified	–	Not specified; 84.3% had post-operative radiotherapy	Not specified	7 (2.9)	–
Kim et al. [25]	94	–	129	Not specified; 98.9% had post-operative radiotherapy	57 (6–155)	1/94 (1.1)	3/129 (2.3)
Damin et al. [26]	38	0	26	Not specified; 86.9% had post-operative radiotherapy	55.8 (34–116)	1/38 (2.6)	0/26 (0)
Wong et al. [27]	58	19	–	Yes (70.7%)	36 (IQR 24–53)	0/58 (0)	–
Piltin et al. [28]	131	Not specified (8 patients with ypNi + and no ALND)	125	Not specified, 78.4% of whole cohort had post-operative radiotherapy	34	1/131 (0.8)	3/125 (2.4)
Choi et al. [29]	84	1	120	Not specified	51 (3–122)	2/84 (2.4)	Not specified
Van Loevezijn et al. [30]	99	Not specified	0	43.4% to level I-III, SCF if PET showed ≥ 4 positive axillary lymph nodes	36 (3.6–64.8)	1/99 (1.0)	–
Kuemmel et al. [31]	94	Not specified	32	Yes (66.4% of the TAD alone group)	43 (95%CI 42–44) from nodal marking	3/119 (2.5) [25 patients were ypN +] 2/94 (2.1)	^1/80 (1.3)
Wu et al. [32]	75	7	93	Around 65% of the cohort	36.6	0/75 (0)	–

^*^SCF supraclavicular fossa

^#^*IMC* internal mammary nodal chain

### Adjuvant treatment

The use of adjuvant systemic treatment was described in eight studies [19–24, 30, 31], and was based on the pathological staging and tumor biology. Hormonal therapy with or without luteinizing hormone-releasing hormone analogs was given to those with HR-positive breast cancers [19–24, 30, 31]. Anti-HER2 adjuvant treatment could be in the form of trastuzumab [19, 21, 23, 31] or trastuzumab emtansine [22, 23]. Further adjuvant chemotherapy has also been described for patients with triple negative breast cancers and residual disease after NST [22, 23, 30].

Eleven studies described the administration of adjuvant radiotherapy [20, 21, 23–28, 30–32] (Table 3). Seven of them specified the use of nodal field irradiation [20, 21, 23, 27, 30–32], which included ipsilateral level I to level III axillary nodes, and supraclavicular lymph nodes with or without the internal mammary nodal chain. Despite nodal pCR in the SLNB or MARI procedure, nodal field irradiation was given to a substantial proportion of patients [20, 21, 27, 30]. Alternative radiotherapy approaches existed in other centers where nodal irradiation was spared in patients with negative SLNs [19, 22].

### Oncological outcomes

**Fig. 2:**
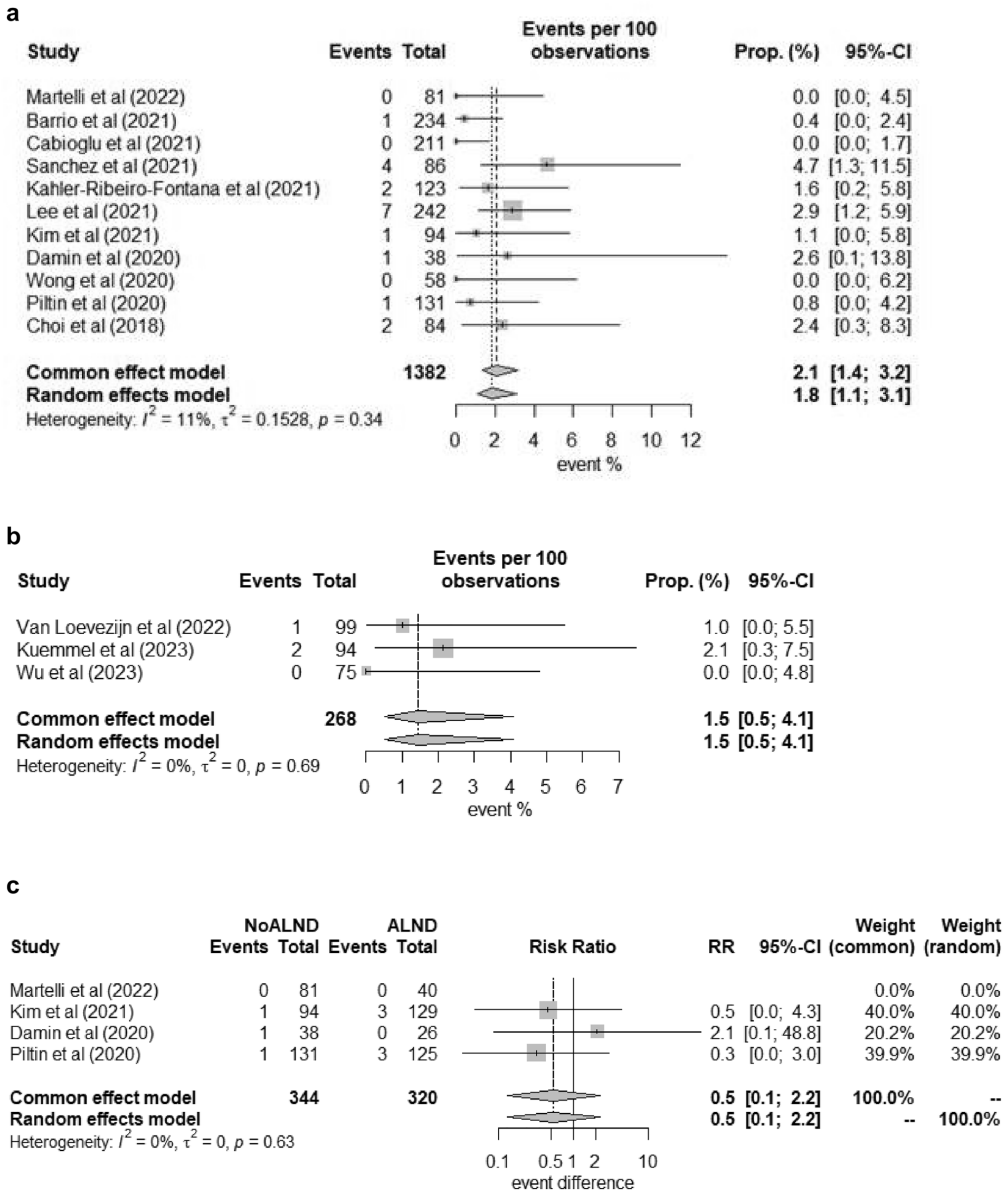
**a** The pooled estimate of axillary recurrence in patients with negative SLNB-alone after NST. **b** The pooled estimate of axillary recurrence in patients with negative MARI/TAD after NST. **c** Forests plots comparing axillary recurrence in patients with negative SLNB-alone and negative ALND

**Fig. 3:**
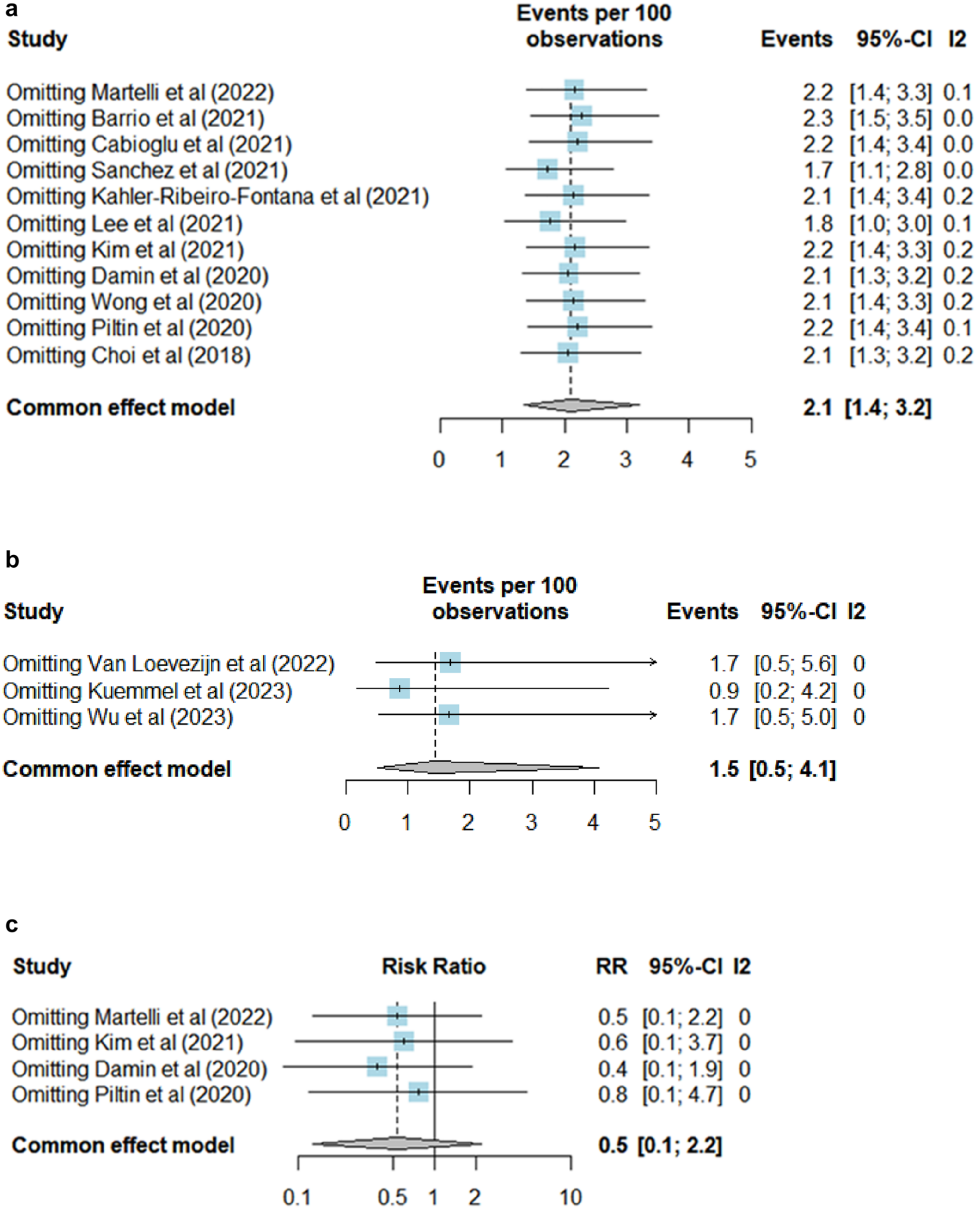
**a** Leave-One-Out Sensitivity Analysis (fixed-effect model) for the pooled estimate of axillary recurrence in patients with negative SLNB-alone after NST. **b** Leave-One-Out Sensitivity Analysis (fixed-effect model) for the pooled estimate of axillary recurrence in patients with negative MARI/ TAD after NST. **c** Leave-One-Out Sensitivity Analysis (fixed-effect model) for comparing axillary recurrence in patients with negative SLNB-alone and negative ALND

Among the 1650 patients with nodal pCR and ALND omission, 1382 patients received SLNB only and there were 19 ipsilateral axillary recurrences (Table 3). No significant heterogeneity was observed among the included studies (*I*^2^ = 11%, *p* > 0.05). The pooled estimate of axillary recurrence in patients with a post-NST negative SLNB and ALND omission was 2.1% (95%CI 1.4–3.2%) (Fig. 2a). The remaining 268 patients who received nodal marking and excision in the form of MARI (99 patients) and TAD (169 patients) were analyzed together. There were 3 ipsilateral axillary recurrences, and the pooled axillary recurrence rate was 1.5% (95%CI 0.5–4.1%) (Fig. 2b). As the 95% confidence interval between the two models highly overlapped, there was no statistical significant difference in the axillary failure rate between SLNB and MARI/TAD. Four studies also described the axillary failure rate among patients with nodal pCR and ALND [19, 25, 26, 28]. There were 3 axillary recurrences among 344 SLNB-only patients and 6 axillary recurrences in the ALND group. The pooled relative risk of SLNB to ALND was 0.5 (95%CI 0.1–2.2) (Fig. 2c). There was no statistically significant benefit of ALND among patients with nodal pCR after NST. Additionally, a Leave-One-Out Sensitivity Analysis has been conducted (Fig. 3a–c), which showed high consistency in meta-estimations. Only mild influence was shown in a few studies.

**Fig. 4:**
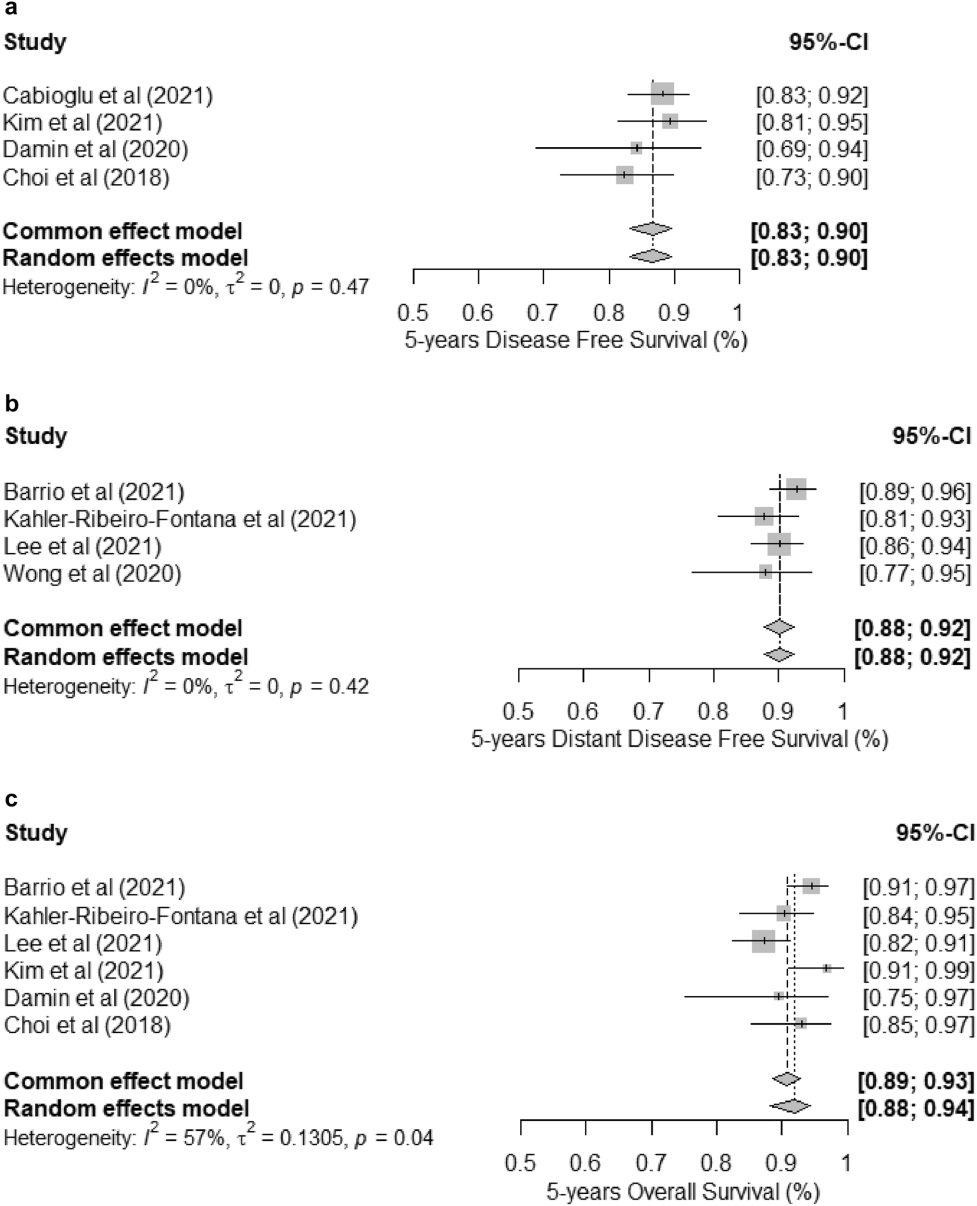
**a** The pooled estimate of 5-year DFS in patients with negative SLNB-alone. **b** The pooled estimate of 5-year DDFS in patients with negative SLNB-alone. **c** The pooled estimate of 5-year OS in patients with negative SLNB-alone

**Fig. 5:**
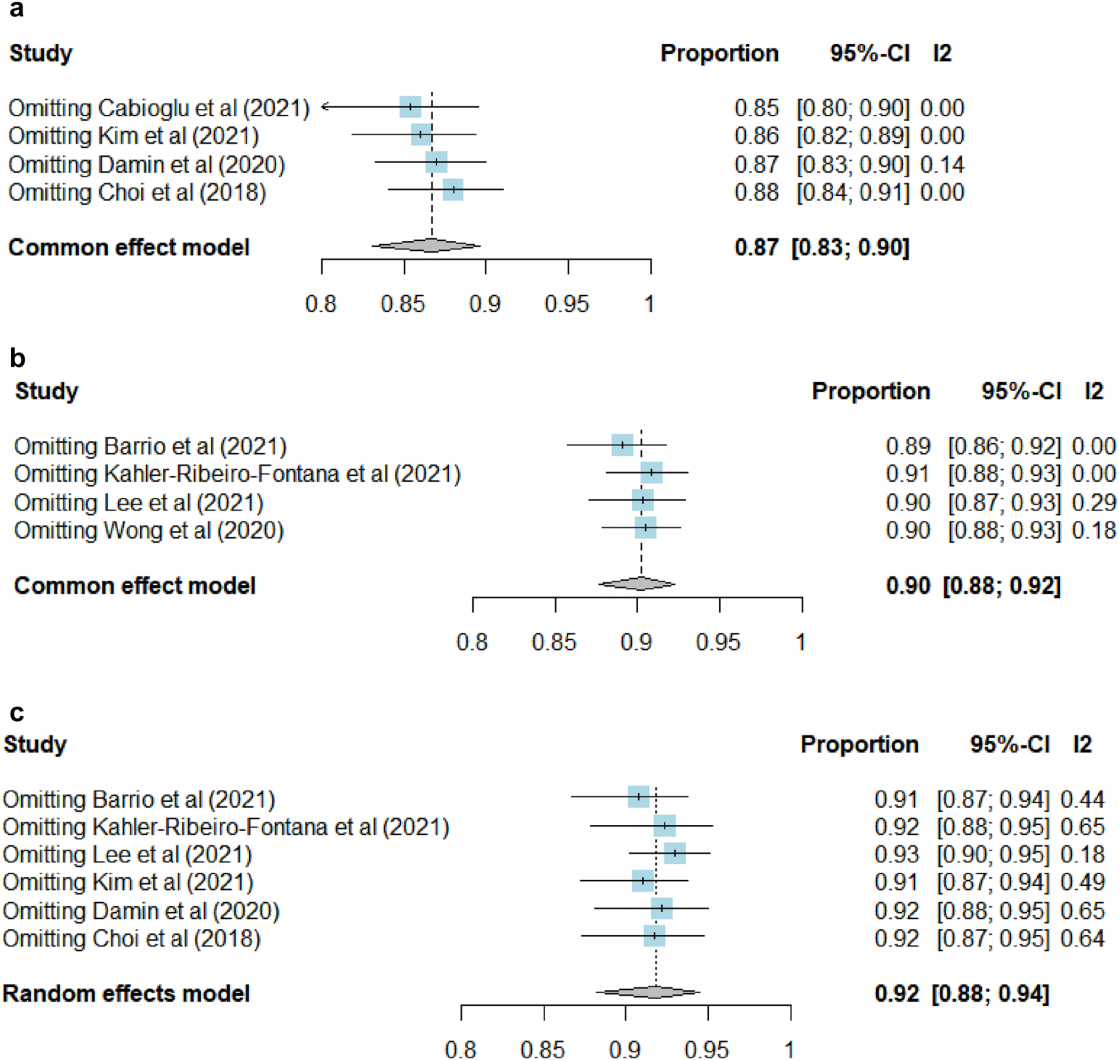
**a** Leave-One-Out Sensitivity Analysis (fixed-effect model) for the pooled estimate of 5-year DFS in patients with negative SLNB-alone. **b** Leave-One-Out Sensitivity Analysis (fixed-effect model) for the pooled estimate of 5-year DDFS in patients with negative SLNB-alone. **c** Leave-One-Out Sensitivity Analysis (random-effect model) for the pooled estimate of 5-year OS in patients with negative SLNB-alone

The 5-year oncological outcome of a negative SLNB-alone was analyzed. The pooled estimate of 5-year DFS was 0.87 (95% CI 0.83–0.90) (Fig. 4a). Four studies evaluated the 5-year DDFS [20, 23, 24, 27] and a total of 63 patients developed distant recurrence in the study period. None of the studies specified the type of distant failure and the pooled estimate of 5-year DDFS was 0.90 (95% CI 0.88–0.92) (Fig. 4b). Heterogeneity was not important among the included studies (*I*^2^ < 40%; *p* > 0.05). Lee et al. [24] and Wong et al. [27] suggested higher risk of distant recurrence among patients with clinical lymph-node metastases at presentation despite pathologically negative nodes after NST. The pooled 5-year OS was 0.92 (95% CI 0.88–0.94) using the random-effect model (Fig. 4c). There was significant heterogeneity among the included studies for OS (*I*^2^ = 57%; *p* = 0.04). Similarly, a Leave-One-Out Sensitivity Analysis has been conducted (Fig. 5a–c). A few studies showed mild influence only on the estimations. Overall, there was high consistency of the meta-estimations and the conclusion remained the same. Only two of the included studies [25, 29] evaluated the oncological outcome of a negative ALND, which demonstrated similar 5-year DFS and OS (Supplementary Fig. 1).

## Discussion

Modern cancer immunotherapy [33] and targeted cancer therapy [34] have revolutionized the systemic treatment landscape of early breast cancers. The remarkably enhanced pCR rate after NST raised concerns about the optimal axillary local treatment. While the role of ALND in axillary staging has waned, the oncological safety of other surgical options is being widely debated [11, 35]. This present meta-analysis demonstrated an extremely low axillary failure rate of SLNB and marked node extirpation in patients with successful nodal conversion following NST. Furthermore, the axillary recurrence rate among patients with negative SLNs was found comparable to those having nodal pCR in completion ALND.

The debate on optimal axillary surgery following NST centered on the accuracy of respective procedures. The relatively higher FNR of SLNB in the neoadjuvant setting led to practical concerns about untreated residual axillary disease, which might jeopardize local cancer control and survival. The retrieval of ≥ 3 SLNs, use of dual tracer SLN mapping, and pre-chemotherapy nodal clipping have been employed to minimize the procedural FNR to below 10% [5–7]. However, the correlation between procedural FNR and long-term survival outcomes was never well established. A multicentre retrospective cohort study published in 2024 evaluated the oncological outcome between SLNB and TAD and found axillary recurrence a rare event regardless of the approach of de-escalation surgery [36]. Martelli et al. [19] published their long-term results of performing SLNB alone in patients achieving nodal pCR. A single mapping agent was used with radiolabeled colloid and a median of two SLNs were harvested. There was no axillary recurrence after a median follow-up of 108 months, and the 10-year DFS and OS were non-inferior to those with ALND. Kahler-Ribeiro-Fontana et al. [23] analyzed 123 patients who became node-negative after NST and underwent SLNB with single tracer. Less than 3 lymph nodes were removed in 74.3% of patients. After a median follow-up of 9.2 years, axillary recurrence occurred in only two patients. Recently, Lim et al. [37] reported their large retrospective series of 314 patients who achieved ypN0 on SLNB-alone. Despite the use of single tracer mapping and the harvest of median two SLNs, the authors demonstrated a better DFS and OS among the SLNB group than the ALND group. These intriguing oncological outcomes of SLNB have inevitably prompted us to reconsider if the proposed surgical refinements were truly mandatory.

The harvest of ≥ 3 SLNs has been proposed to minimize the FNR, but technically was not always feasible. After NST, adequate mapping was only noted in 34% of patients in the SENTINA study [5] and half of the patients in the ACOSOG Z1071 trial [7]. It remained disputable if patients with inadequate SLN mapping warrant a completion ALND. Pfob et al. [38] looked into the oncological outcomes of different axillary surgery based on the data from the German Cancer registry. No significant difference in invasive disease-free survival was observed among patients receiving ALND, SLNB with < 3 SLNs, SLNB with ≥ 3 SLNs, and TAD. Sharp et al. [39] proposed a trend of better recurrence-free survival (RFS) with ≥ 2 SLNs. Among the 68 patients who were converted to node-negative breast cancer, 47% had one or two negative SLNs localized by a single mapping agent. There were a total of two axillary recurrences with the harvest of 3 and 6 SLNs, respectively. Pre-chemotherapy nodal marking could potentially increase the number of lymph nodes excised during definitive surgery. However, failure of TAD happened in clinical practice due to failure in clipped node or SLN localization. Kim et al. [40] overcame this difficulty with the use of fluoroscopy and cone-beam computed tomography during wire localization, at a cost of extra radiation. Other commercially available devices for marking axillary lymph nodes have also been described. Controversies also exist regarding the ideal number of positive nodes to be marked. Lim et al. [41] examined the accuracy of clipped nodes in the prediction of axillary status following NST. Fourteen patients received nodal clipping to one to three malignant nodes. The FNR was 7.1% when one clipped node was excised, i.e., the MARI procedure [8]. When the second clipped node was excised, the FNR dropped to zero. The optimal number of TAD nodes is similarly uncertain. Simons et al. [42] compared the diagnostic accuracy of RISAS, MARI, and SLNB in their prospective multicenter trial. When both SLNB and marked node extirpation were successful, a minimum of 2 lymph nodes were adequate to achieve an FNR of 2.5%. Their finding was reciprocated in the SenTa study [43], in which no false-negative event occurred in patients with 2 or more TAD nodes. Nevertheless, these findings were based on the evaluation of procedural FNR as primary study outcome and correlation with oncological outcome is yet to be demonstrated.

Standardization in the selection of patients for axillary surgical de-escalation has yet to be achieved. Pre-chemotherapy tumor and nodal staging varied among the included studies. While procedural FNR of SLNB did not appear to be influenced by the presenting nodal staging [6, 7], the analysis was based on small cohorts of patients with high axillary nodal burden. Pre-chemotherapy N2 breast cancers constituted only around 5% of patients in the ACOSOG Z1071 [7] and SN FNAC study [6]. When 4 or more abnormal nodes were detected in initial axillary ultrasound, Caudle et al. [9] found a higher chance of discordance between clipped node and SLN, potentially increasing the FNR of SLNB to 19% in the ACOSOG Z1071 trial [7]. Although TAD has shown to be a reliable surgical technique among patients with initial node-positive breast cancers, its application in locally advanced nodal disease is also limited. The Chinese group found a higher procedural FNR of TAD when 24 patients with cN2-3 disease were included in their analysis [32]. Three false-negative events were noted among 58 patients with N2-3 disease in the evaluation of the RISAS procedure [42]. It remained questionable if pre-NST nodal burden would influence the decision of axillary surgery, adjuvant radiotherapy, or subsequent long-term survival. Furthermore, while several international guidelines triaged patients to axillary de-escalation based on the post-NST clinical nodal status [44, 45], the definition of clinically negative nodal status has not been standardized. Physical examination and/or AUS are the commonly used assessment modalities. MRI and PET have also been studied, but all modalities demonstrated suboptimal positive and negative predictive value in the evaluation of axillary response after NST [46].

Untreated axillary tumor residue is probably expected in the de-escalation of axillary surgery. In the ACOSOG Z0011 [47] and AMAROS trial [48], approximately 30% of patients in the ALND group had additional metastatic non-SLNs. The extremely low axillary recurrence rate and non-inferior DFS in these studies suggested the therapeutic significance of adjuvant whole breast radiotherapy (with or without regional nodal irradiation) and systemic treatment in patients with low axillary nodal burden. However, the role of locoregional irradiation in patients who achieved post-NST nodal pCR after NST has not been elucidated. While adjuvant full nodal irradiation was used in some centers [20, 21], Martelli et al. [19] found zero axillary recurrence in their series with whole breast irradiation only. Pre-chemotherapy axillary nodal burden may contribute to the decision of nodal field irradiation. Schlafstein et al. [49] identified 1963 clinical N1 patients with successful nodal conversion on SLNB from the National Cancer Database and found no added overall survival benefit with regional nodal irradiation. Similarly, the Dutch group personalized the use of adjuvant nodal irradiation to patients with high nodal burden in pre-chemotherapy positron emission tomography–computed tomography (PET–CT) scan [30]. In their series, only 1% of the selected patients suffered from axillary recurrence with the omission of both ALND and axillary radiotherapy. Some researchers further extended the omission of adjuvant regional nodal irradiation to patients with low residual volume nodal diseases and demonstrated a comparable overall survival rate [50]. The phase III randomized clinical trial NSABP B-51 has recently finished accrual, and their results would better define the role of adjuvant nodal field irradiation in post-NST patients with nodal pCR [51].

There are several limitations to this meta-analysis. Most included studies were retrospective with between-study heterogeneity. The presenting clinical staging, definition of pre-NST nodal positivity, and post-NST nodal conversion varied. The extent of adjuvant radiotherapy was also different. With the low incidence of axillary recurrence, we are unable to determine if these heterogeneities contribute to any adverse oncological outcome. It is also difficult to portray the patient population who benefits most from this change in practice. Furthermore, the actual survival time of each studied individual was not available. Survival data were calculated based on odd ratios of events at a particular time point without taking into account the censored population [52], potentially resulting in over-estimation of treatment effect. Finally, MARI and TAD are relatively new techniques, and we are only beginning to see the short-term survival data of marked node extirpation from expert centers worldwide. However, direct comparison between different treatment modalities for long-term oncological outcome has not yet been feasible. The multi-national prospective AXSANA study, with a target accrual of 4500 participants, would hopefully address the uncertainties in de-escalation of axillary treatment [53].

## Conclusion

This meta-analysis demonstrated an extremely low axillary recurrence rate with SLNB and MARI/TAD in patients who have achieved pCR after NST. The 5-year DFS, DDFS, and OS are excellent in good responders despite the omission of ALND. However, long-term oncological data are necessary, in particular for the MARI/TAD procedures, to identify the optimal axillary treatment. Standardization of the pre-chemotherapy and pre-operative workup are also essential in triaging suitable patients for surgical de-escalation.

## Supplementary Information

Below is the link to the electronic supplementary material.

Supplementary file1 (DOCX 41 KB)
