# Supplementary figures and images for: Different strategies in de-escalation of axillary surgery in node-positive breast cancer following neoadjuvant treatment: a systematic review and meta-analysis of long-term outcomes
Source: Breast Cancer. 2025 Apr 5;32(4):638–53. doi: 10.1007/s12282-025-01692-9 (PMC12174186; doi:10.1007/s12282-025-01692-9)

Supplementary Fig 1 The pooled estimate of 5-year DFS and OS in patients with negative ALND


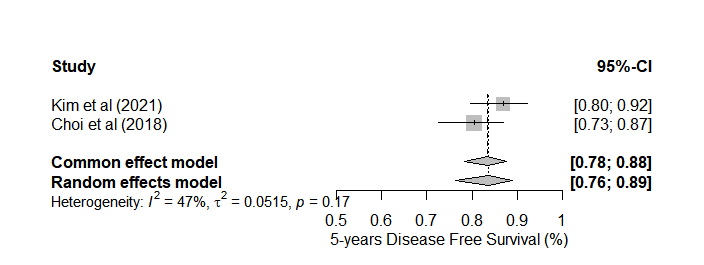


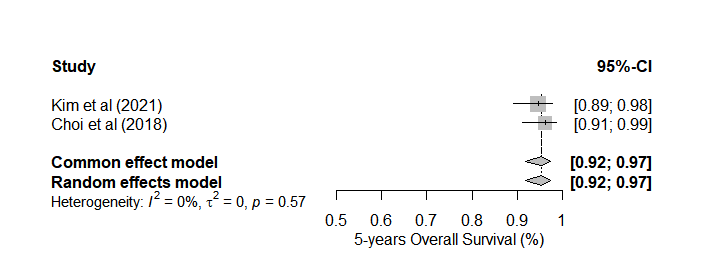

Supplement: Supplementary file 1 — Supplementary file1 (DOCX 41 KB) [file 12282_2025_1692_MOESM1_ESM.docx]
